# Metformin induces apoptosis in mesenchymal stromal cells and dampens their therapeutic efficacy in infarcted myocardium

**DOI:** 10.1186/s13287-018-1057-0

**Published:** 2018-11-08

**Authors:** Xiao He, Meng-Wei Yao, Ming Zhu, Dong-Lan Liang, Wei Guo, Yi Yang, Rong-Seng Zhao, Ting-Ting Ren, Xiang Ao, Wei Wang, Chun-Yu Zeng, Hua-Ping Liang, Dong-po Jiang, Jian Yu, Xiang Xu

**Affiliations:** 1 Department of Stem Cell and Regenerative Medicine, State Key Laboratory of Trauma, Burn and Combined Injury, Daping Hospital and Research Institute of Surgery, Army Medical University, Chongqing, People’s Republic of China; 2 First Department, State Key Laboratory of Trauma, Burn and Combined Injury, Daping Hospital and Research Institute of Surgery, Army Medical University, Chongqing, People’s Republic of China; 3 Department of Biochemistry and Molecular Biology, College of Basic Medical Sciences, Army Medical University, Chongqing, People’s Republic of China; 4 Department of Histology and Embryology, Qingdao University Medical College, Qingdao, Shandong People’s Republic of China; 5 Department of Cardiology, Daping Hospital and Research Institute of Surgery, Army Medical University, Chongqing, People’s Republic of China; 6 Department of Critical Care Medicine, Daping Hospital and Research Institute of Surgery, Army Medical University, Chongqing, 400042 People’s Republic of China; 7 Department of Pathology of Pittsburgh Cancer Institute, Pittsburgh, PA USA

**Keywords:** Mesenchymal stromal cells, Metformin, Myocardial infarction, Apoptosis, Diabetes mellitus

## Abstract

**Background:**

Cardiovascular complications, especially myocardial infarctions (MIs), are the leading mortality cause in diabetic patients. The transplantation of stem cells into damaged hearts has had considerable success as a treatment for MI, although whether antidiabetic drugs affect the therapeutic efficacy of stem cell transplantation is still unknown. This study aims to understand whether and how metformin, one of the first-line drugs used to treat type 2 diabetes mellitus (T_2_DM), induces mesenchymal stromal cell (MSC) apoptosis and dampens their cardioprotective effect after transplantation into infarcted hearts.

**Methods:**

A mouse MI model was generated via permanent ligation of the left anterior descending (LAD) coronary artery. MSCs with or without metformin treatment were transplanted after MI in diabetic mice. Echocardiography was used to assess cardiac function and determine cardiac remodeling, and TTC staining was performed to evaluate infarction size. A mouse gavage model was performed to evaluate bone marrow MSCs for flow cytometry assay.

**Results:**

Metformin dampened MSC therapeutic efficacy, which increased infarct size and restricted functional cardiac recovery. Specifically, metformin induced the activation of AMP-activated protein kinase (AMPK)-mediated apoptosis through the inhibition of S6K1-Bad-Bcl-xL cell survival signaling, resulting in the upregulated expression of apoptosis-associated proteins and increased MSC apoptosis. Accordingly, counteracting AMPK attenuated metformin-induced apoptosis in MSCs and partially restored their cardioprotective effects in diabetic mice with MI. Furthermore, a decrease in peripheral blood MSCs was found in patients with T_2_DM who had a metformin medication history.

**Conclusions:**

Our results highlight an unexpected adverse effect of metformin-induced MSC apoptosis through AMPK-mediated mTOR suppression, which is attenuated by an AMPK inhibitor. Moreover, AMPK inhibition may be a novel strategy for enhancing the effectiveness of stem cell therapy after MI in diabetes.

**Electronic supplementary material:**

The online version of this article (10.1186/s13287-018-1057-0) contains supplementary material, which is available to authorized users.

## Background

Cardiovascular complications, especially myocardial infarction (MI), are the leading cause of death in diabetic patients [1, 2]. Recent studies reported that injected stem cells can salvage the myocardium from death through a protective paracrine mechanism and that several types of stem cells have been tested for safety and efficacy in animal models of MI and human MI patients [3, 4].

Mesenchymal stromal cells (MSCs), which contain a subpopulation of stem cells able to differentiate in bone, cartilage, and fat, are one of the most promising candidates for stem cell therapy for heart disease. MSCs exist in most tissues in the body including the umbilical cord, bone marrow, adipose tissue, and peripheral blood; exhibit a strong capacity for replication in vitro; express CD105, CD90, and CD73; express low levels of MHC-I; and lack expression of MHC-II, CD11b, CD14, CD34, CD45, and CD31 [5]. The isolation of MSCs according to the International Society for Cellular Therapy (ISCT) criteria has produced heterogeneous, nonclonal cultures of stromal cells containing stem cells with different multipotent properties, committed progenitors, and differentiated cells [6]. MSCs in situ are speculated to have an indispensable role in tissue homeostasis by replacing dysfunctional or dead cells [7]. Recently, our group found that the number of circulating mesenchymal-like cells (CD34−/CD105+) is significantly decreased in type 2 diabetes mellitus (T_2_DM) patients and is negatively correlated with the progression of chronic diabetic complications [8]. In addition, our group reported that the transplantation of MSCs into damaged hearts has a therapeutic effect on MI and that prolyl hydroxylase domain protein 2 (PHD2) silencing could enhance the protective effect of MSCs on cardiomyocytes [9]. Since cardiovascular complications, especially MI, greatly rely on MSCs to regenerate cardiac tissue and promote a functional recovery, it is of great importance to determine whether antidiabetic drugs impact the therapeutic efficacy of stem cell transplantation in patients with diabetes mellitus. Moreover, because diabetic patients are chronically exposed to antidiabetic drugs, this long-term exposure and drug accumulation resulting from the compromised renal and hepatic functions commonly seen in these patients may amplify the toxic effects of antidiabetic drugs [10]. Therefore, understanding the effects of antidiabetic agents on stem cell biology is indispensable for the development of stem cell-based therapy for treating diabetic patients with MI.

Few studies have investigated the effects that antidiabetic drugs have on stem cells. Some have suggested that antidiabetic agents affect the multipotency and proliferation of MSCs. Benvenuti et al. and Beck et al. reported that thiazolidinediones (TZDs) stimulate adipogenesis and decrease osteoblastogenesis in human MSCs [11, 12]. On the other hand, insulin can promote the proliferation and osteogenic differentiation of human MSCs [13]. In addition, metformin has previously been shown to promote the osteogenic differentiation and inhibit the adipogenic differentiation of MSCs [14]. However, little is known about the effect of antidiabetic agents on the therapeutic efficacy of stem cell transplantation in diabetic patients with MI. To study the effects of antidiabetic drugs on the therapeutic efficacy of stem cell transplantation, we screened common clinical antidiabetic agents such as various insulin analogs, TZDs, and metformin in vitro. Specifically, we assessed the proliferation and apoptosis of MSCs after treatment with these drugs. We discovered that metformin, which is recommended by the American Diabetes Association (ADA) as a first-line hypoglycemic treatment for T_2_DM, clearly induced MSC apoptosis. To clarify the correlation between metformin and stem cell transplantation and to reveal the mechanism underlying our preliminary observations, we further performed a series of in vitro and in vivo experiments. Here, we report that metformin dampens MSC therapeutic efficacy, which increased infarct size and restricted functional cardiac recovery, through inducing MSC apoptosis in an AMPK-mTOR pathway-dependent manner. Additionally, AMPK inhibition may be a novel strategy for enhancing the effectiveness of stem cell therapy after MI in diabetes.

## Methods

### Human samples

Blood samples were obtained from the Hypertension and Endocrinology Department of the Third Affiliated Hospital, Third Military Medical University, after Ethics Committee approval based on informed consent from patients. All participants gave written consent before participation in the study. The investigation conforms to the principles outlined in the Declaration of Helsinki. Upon arrival in the laboratory, the samples were immediately processed for the detection of hMSCs by flow cytometry, as described in the protocol (hMSC Analysis Kit, BD Stemflow, USA).

### Cells

Human umbilical cords (*n* = 5) were collected from full-term women immediately after cesarean section in the Gynaecology Department of the Third Affiliated Hospital, Army Military Medical University of China, after approval was obtained from the Institutional Ethics Committee. After vessel removal, the cords were cut into pieces with scissors and cultured in α-MEM (10% FBS, 2 mM L-glutamine). All MSCs used in the experiment were derived from passages 5–10. The MSCs were used after thorough characterization by flow cytometry (hMSC Analysis Kit, BD Stemflow, USA), as defined by the International Society for Cellular Therapy. After incubation in differentiation media, the MSCs differentiated into osteocytic, chondrocytic, and adipocytic lineages (Additional file 1: Figure S1).

### Cell apoptosis analysis

To quantify the apoptotic MSCs, cells were harvested at 24 h, 48 h, and 72 h after treatment with different concentrations of metformin with or without compound C (1 μM) and siAMPK in α-MEM (10% FBS, 2 mM L-glutamine). To quantify apoptosis, cells were washed and stained with an annexin V-FITC and propidium iodide (PI) apoptosis kit (KeyGen Biotech, China) according to the manufacturer’s instructions. Stained cells were analyzed by flow cytometry (NovoCyteTM, ACEA Biosciences, USA), and the data were analyzed using NovoExpress V.1.2.1 software.

### Cell cycle analysis

Cell cycle analyses were performed using Cell Cycle Analysis Kits (KeyGen Biotech, China) according to the manufacturer’s instructions. A flow cytometer (NovoCyteTM, ACEA Biosciences, USA) was used to analyze cells and to determine the percentage of cells that were in the G0/G1, S, and G2/M phases of the cell cycle using NovoExpress V.1.2.1 software.

### Western blot

MSCs were lysed in ice-cold lysis buffer on ice. A protein quantification of cell lysates was completed using a Bradford assay (Bio-Rad, Hercules, CA). Equal quantities of protein were separated by 8–12% SDS-PAGE and transferred to a nitrocellulose membrane. The membranes were blocked with 5% nonfat milk in TBST solution (0.05% Tween 20 in Tris-buffered saline) and then incubated overnight at 4 °C with primary antibodies. The blots were washed with TBST, incubated with the appropriate horseradish peroxidase-conjugated secondary antibodies (1:1000) for 2 h at room temperature, washed again with TBST, and then developed with ECL western blotting substrate (Thermo Scientific, Waltham, MA). The following antibodies were purchased from CST: AMPK, phospho-AMPK, Akt, phospho-S473-Akt, phospho-T308-Akt, mTOR, phospho-mTOR, S6K1, phospho-S6K1, RagB, LC3B, Bad, phospho-Bad, Bcl-xL, cleaved-caspase 3, and β-actin.

### Mice

A genetic mouse model of diabetes B6.BKS(D)-*Lepr*^db^/J (Jackson Laboratory, Bar Harbor, ME, USA) was bred in the Experimental Animal Center of Third Affiliated Hospital, Third Military Medical University; the detailed methods for generating the diabetic myocardial infarction mouse model followed those described in our previous study [8]. The optimal oral metformin dosage for many diabetic patients is ~ 2 g/day. The human metformin dosage of 20 mg/kg/day orally translates to an equivalent mouse dosage of 250 mg/kg/day, based on the normalization to body surface area [15]. In this experiment, the 8- to 12-week-old male diabetic mice were treated with saline, metformin (250 mg/kg/day, i.g., Sigma), or metformin + compound C (0.1 mg/kg/day, i.g., Sigma) decoction by gavage for 4 weeks. Afterward, all the diabetic mice were sacrificed to isolate bone marrow MSCs for flow cytometry assay. A permanent ligation of the left anterior descending (LAD) coronary artery and cell injection were performed in male diabetic mice (8 weeks old). Immediately after coronary ligation, 1 × 10^5^ MSCs suspended in 50 μL PBS were injected at five sites in the anterior and posterior infarct border zones of the ischemic myocardium. FK506 (3 mg/kg/day, i.p., Sigma) was administered to MSC-transplanted and sham-operated animals daily from 1 week before the operation until the end of the study. Metformin (250 mg/kg/day, 4 weeks, i.g., Sigma) or metformin + compound C (0.1 mg/kg/day, 4 weeks, i.g., Sigma) was administered to LAD-operated animals daily from the day of operation until 4 weeks after operation. Animal care and all experimental procedures were performed in strict accordance with the approved protocols and animal welfare regulations of the Animal Care and Use Committee at the Third Military Medical University and EU (Directive 2010/63/EU) ethical guidelines and comply with the National Institutes of Health guide for the care and use of laboratory animals. Echocardiography was used to assess the cardiac function and determine cardiac remodeling. TTC staining was performed to evaluate infarction size.

### Real-time cell proliferation monitoring

MSCs were seeded at densities of 5 × 10^3^ cells/well into an E-plate 16 (ACEA Biosciences, San Diego, CA) containing 100 μL medium per well and monitored on an xCELLigence Real-Time Cell Analyzer Dual Plate (RTCA DP) instrument (ACEA Biosciences). When the cells entered the log phase, 2 mM metformin, 2 mM metformin + 1 μM compound C, or 2 mM metformin + siAMPK was added in α-MEM (10% FBS, 2 mM L-glutamine). The cells were treated for 160 h and incubated at 37 °C in a 5% CO_2_ atmosphere. RTCA software v. 1.2.1 was used to record the cell index (CI). All experiments were repeated at least three times.

### Flow cytometry (FCM) analysis

For live cell counting, transplanted MSCs were labeled with CM-DiI (CellTracker™ CM-DiI Dye, Invitrogen, Thermo Fisher, USA) according to the manufacturer’s instructions, and hearts with MI were digested. Dissociated cells were separated by filtering through a 30-μm filter. Most of the cardiomyocytes (> 30 μm diameters) were discarded, and the small cell fraction (< 30 um) was collected. The surviving CM-DiI (PE+) cells were identified with fluorescence microscopy, and the number of CM-DiI (PE+) cells was counted with NovoExpress V.1.2.1 software.

### Statistical analysis

Unless specified otherwise, all experiments were repeated at least three times. Data are presented as the means ± SD. Statistical analysis (ANOVA and Student’s *t* test) was performed using SPSS 13.0 software; *p* < 0.05 was considered to indicate statistical significance.

## Results

### Metformin dampens MSC therapeutic efficacy in MI

**Fig. 1:**
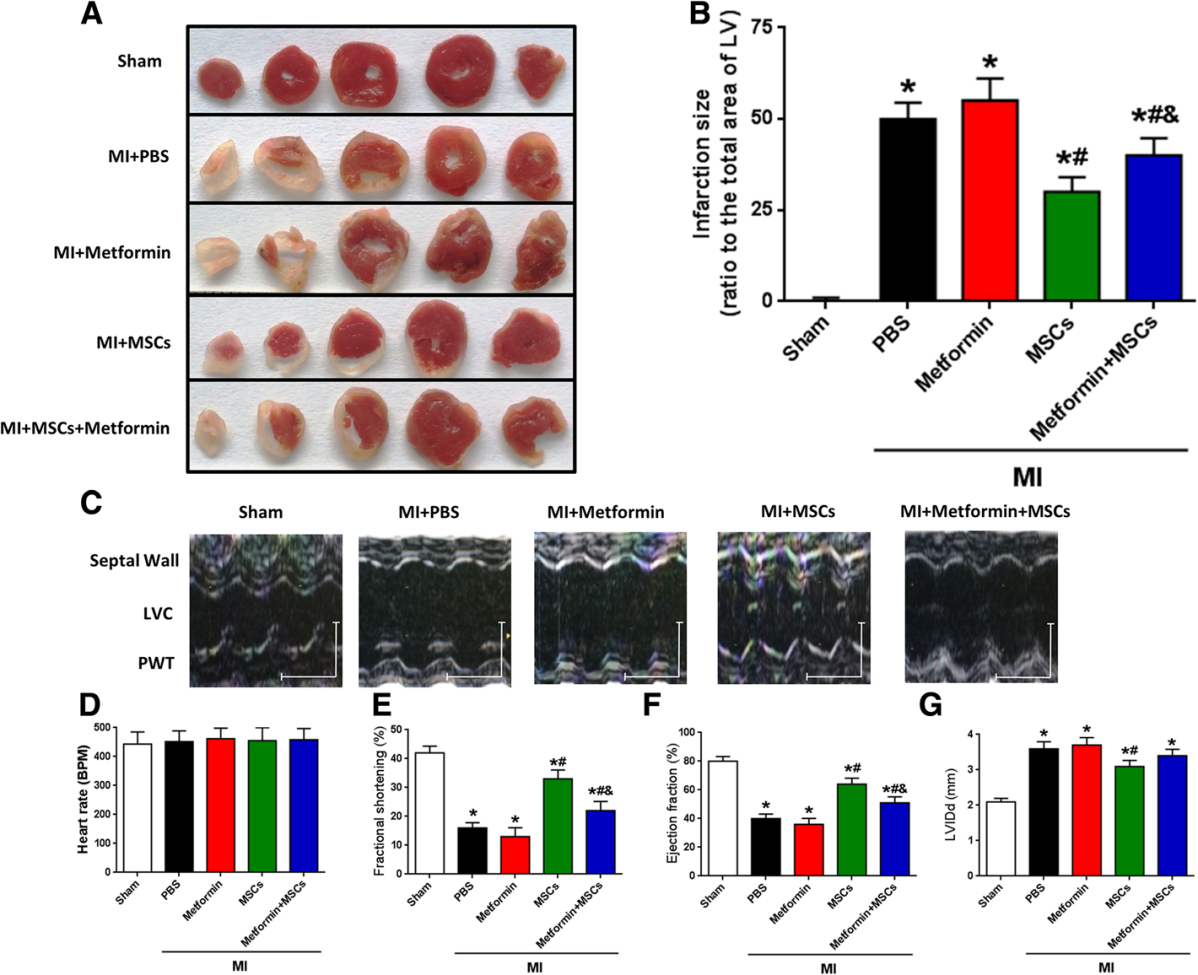
Metformin dampens MSC therapeutic efficacy in MI. **a** Representative images of TTC-stained heart sections obtained from Sham, MI + PBS, MI + metformin, MI + MSCs, and MI + metformin + MSCs groups at 4 weeks after MI. **b** LV infarct sizes expressed as the ratio of the area of the infarct band to the total area of LV (*n* = 23). **c** Representative M-mode images of hearts with sham surgery or MI at 4 weeks after PBS, metformin, MSCs, and metformin + MSCs treatment. Scale bar: *X* axis: 0.1 s; *Y* axis: 0.2 cm. **d** Heart rates were controlled to be similar in different groups (*n* = 23). **e**–**g** LV fraction shortening (**e**), LV ejection fraction (**f**), and diastolic left ventricle internal diameter (LVIDd) (**g**) at 4 weeks after treatment (*n* = 23). *p < 0.05 vs. sham; ^#^*p* < 0.05 vs. MI + metformin; ^&^*p* < 0.05 vs. MI + MSCs, by one-way ANOVA

To determine whether taking metformin impairs the therapeutic effect of stem cells, we evaluated the effects of MSC transplantation on infarct size and cardiac function in post-MI diabetic mouse hearts [9]. Myocardial infarct size was significantly smaller in diabetic mice treated with MSCs than in those treated with PBS or metformin at 4 weeks post-MI, whereas the metformin + MSCs group showed that metformin dampens the MSC therapeutic efficacy in MI (Fig. 1a, b). Although the infarction size in the metformin group was larger than that in the PBS group, there was no significant difference (*p* = 0.2453). These data suggest that the regeneration of cardiac tissue after MSC transplantation was limited by metformin treatment. In line with the histologic change, the left ventricle ejection fraction (EF) and fractional shortening (FS) of the post-MI hearts at 4 weeks were lower in diabetic mice treated with metformin + MSCs than in those treated with MSCs alone (Fig. 1c, e, and f). MI significantly decreased the EF and FS in all groups of post-MI hearts compared to sham-operated hearts (Fig. 1e, f). Left ventricle dilation induced by MI injury was confirmed with diastolic left ventricle internal diameter (LVIDd) of post-MI hearts at 4 weeks, which was significantly larger than that of the sham group. However, MSC but not MSC + metformin transplantation significantly limited the increase in LVIDd induced by MI (Fig. 1g). These results indicate that MSC transplantation reduces the post-MI deterioration of cardiac function and that this cardioprotective effect is impaired by metformin treatment in diabetic mice.

### Metformin induces MSC apoptosis

**Fig. 2:**
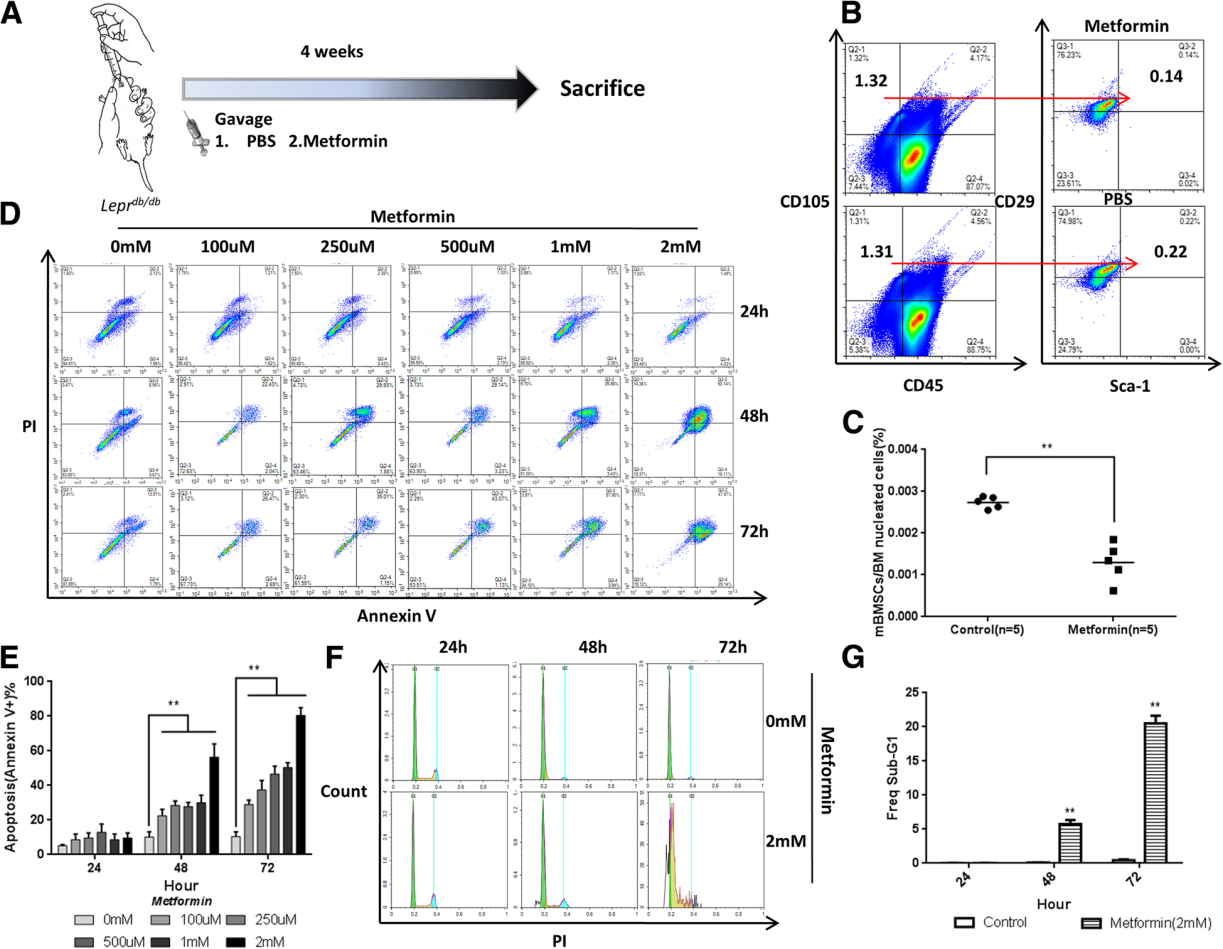
Metformin induces MSC apoptosis. **a** Diabetic mice were administered with PBS or metformin by gavage for 4 weeks, and then all mice were sacrificed to isolate mouse bone marrow mesenchymal stem cells (mBMSCs) for flow cytometry assay. **b**, **c** In vivo effects of metformin on mBMSCs. Compared with the PBS group, metformin decreased the number of mBMSCs (CD45−/CD105+/CD29+/Sca-1+). Lines in **c** represent the mean. **p < 0.01, by *t* test, *n* = 10. mBMSC, mouse bone marrow mesenchymal stromal cell. **d**, **e** Human umbilical cord MSC apoptosis ratio (annexin V+) induced by different concentrations of metformin at 24 h, 48 h, and 72 h. **f**, **g** Comparison of cell cycle distribution between the metformin group and control group by flow cytometry. In **e**, **c**, and **g**, **p < 0.01 by one-way ANOVA (*n* = 3 per group in **d** and **f**)

To directly test whether endogenous MSCs were affected by metformin in the animal model, we evaluated the CD45−/CD105+/CD29+/Sca-1+ cells in the bone marrow of diabetic mice treated with saline or metformin (250 mg/kg/day) decoction by gavage for 4 weeks. After treatment with saline or metformin (250 mg/kg/day) decoction by gavage for 4 weeks, the diabetic mice were sacrificed to isolate the bone marrow for the flow cytometry assay (Fig. 2a). As expected, metformin treatment led to a decrease in the number of diabetic mouse bone marrow MSCs (CD45−/CD105+/CD29+/Sca-1+) (Fig. 2b, c). Together, these results show that metformin caused a decrease in the endogenous MSC levels in diabetic mice.

To understand how metformin decreases the levels of MSCs, we monitored apoptosis, autophagy, and proliferation inhibition. Given that most current in vitro studies of metformin drug concentration use mM levels [16], which are far greater than the physiological dose [17], we decided to start from the μM-class drug concentration, which is close to the physiological dose, in the following experiments. Neither autophagy nor proliferation were affected by treatment, but metformin greatly increased the rate of apoptosis (annexin V+) of human umbilical cord-derived mesenchymal stromal cells (hUC-MSCs) in vitro in a dose- (100 μM, 250 μM, 500 μM, 1 mM, 2 mM) and time-dependent manner (Fig. 2d, e), suggesting that metformin induces apoptosis in hUC-MSCs. Incubation with 2.0 mM metformin for 48 h or 72 h increased the rate of apoptosis to 71.3% or 89.2%, respectively.

In addition to MSCs, there are cardiomyocytes and myocardial blood vessels that could affect the recovery of cardiac function after MI. Thus, we studied the effects of metformin on mouse cardiomyocytes and human endothelial cells. However, metformin had no such effect on cardiomyocytes and endothelial cells (Additional file 1: Figure S2A-S2D). Moreover, metformin also had no such effect on adult cells such as fibroblasts (Additional file 1: Figure S2E and S2F).

To quantify the magnitude of metformin-induced apoptosis in MSCs, we studied the effects of metformin on cell cycle distribution and progression. As shown in Fig. 2c, d, compared to the control, metformin induced a sub-G1 peak of approximately 20% at 72 h, which is suggestive of apoptosis.

### Metformin induces the activation of AMP-activated protein kinase (AMPK)-mediated apoptosis

**Fig. 3:**
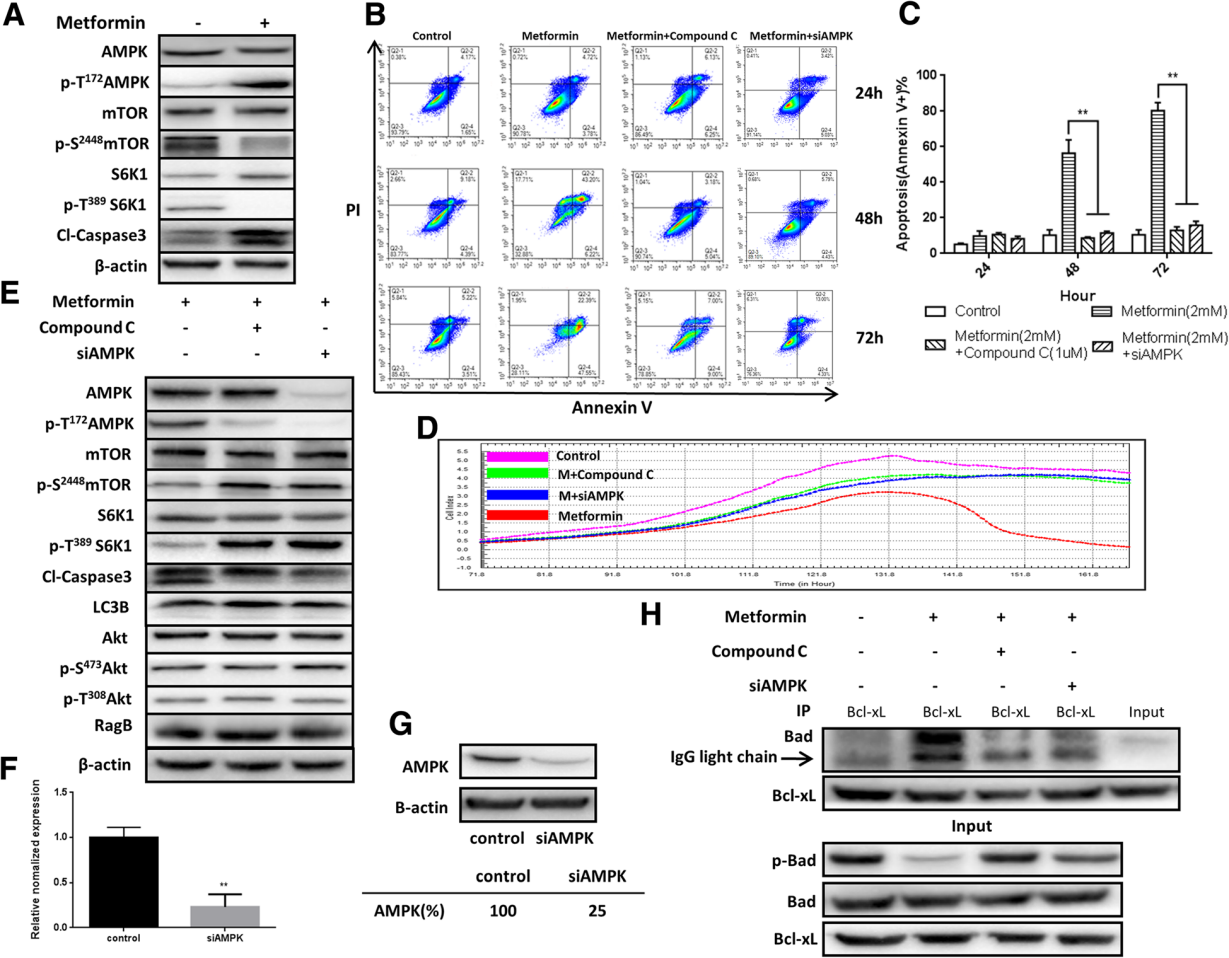
Metformin induces the activation of AMP-activated protein kinase (AMPK)-mediated apoptosis. **a** AMPK, mTOR, S6K1, their phosphorylated forms, and cleaved-caspase 3 were assessed by western blot analysis after treatment with or without metformin (2 mM). β-actin was used as loading control. **b**, **c** Treatment with an AMPK inhibitor (compound C) (1 μM) and siAMPK significantly blocked metformin-induced hUC-MSC apoptosis. Bars in **c** represent the mean ± SD (*n* = 3 per group). **p < 0.01, by one-way ANOVA. **d** Real-time cellular analysis (RTCA) of compound C and siAMPK on metformin-induced hUC-MSC apoptosis. **e** Compound C and siAMPK significantly inhibited metformin-induced AMPK phosphorylation but have no effect on LC3B, Akt, and Rag B. β-actin was used as loading control. **f**, **g** Expression of AMPK in hUC-MSCs 24 h after transient transfection of siAMPK at the transcriptional (qRT-PCR, **p < 0.01, by *t* test, *n* = 3) and translational (western blot) level. **h** hUC-MSCs were treated with metformin (2 mM) with or without compound C (1 μM) and siAMPK for 36 h. After treatment, cell lysates were immunoprecipitated with an anti-Bcl-xL antibody and immunoblotted with an anti-Bad antibody. The presence of Bad and Bcl-xL in the lysates was examined. The results are representative of three independent experiments

Metformin has previously been shown to inhibit the growth of breast cancer cell lines in vitro via AMPK induction and mTOR inhibition [18]. To determine whether a similar mechanism is involved in metformin-induced MSC apoptosis, we explored the AMPK-mTOR signaling pathway. As shown in Fig. 3a, metformin treatment activated AMPK and suppressed mTOR and its downstream effector S6K1, a signaling pathway critical for cell proliferation and survival. Furthermore, the metformin-induced hUC-MSC apoptosis was confirmed by the increased levels of cleaved-caspase 3 (Fig. 3a).

Next, we investigated whether a specific inhibitor of AMPK, compound C, and specific siAMPK (Fig. 3f, g) reverse the inhibitory effects of metformin on MSC survival. As shown in Fig. 3b, c, treatment with compound C (1 μM) and siAMPK significantly inhibited metformin-induced MSC apoptosis, AMPK activation, and mTOR and S6K1 suppression (Fig. 3e). In support of this evidence, real-time cellular analysis (RTCA) also showed decreased metformin-induced apoptosis in hUC-MSCs when treated with compound C and siAMPK for 160 h (Fig. 3d).

In addition to AMPK, the mTOR pathway is also regulated by Akt, which belongs to a family of lipid kinases critical for cell growth, survival, and proliferation [19]. To explore which pathway is the main regulator of mTOR, we explored the Akt- and AMPK-mTOR signaling pathways. As shown in Fig. 3e, treatment of MSCs with an AMPK inhibitor reversed metformin-activated AMPK, which suppressed mTOR and its downstream effector S6K1. However, no significant change in Akt was observed.

In addition to apoptosis-related caspase 3, autophagy-related LC3B was also considered. However, the level of LC3B was not significantly different between groups (Fig. 3e), and metformin-induced MSC apoptosis was not changed after treatment with an autophagy inhibitor (3-methyladenine, 3-MA). In addition, metformin treatment did not change the level of Rag B, which was reported to be involved in mTOR inhibition by metformin [20].

S6K1 signals cell survival and cell growth through the phosphorylation of substrates such as the Bcl-2 family member Bad and the ribosomal subunit S6, respectively [21, 22]. Treatment with metformin significantly reduced Bad phosphorylation (Fig. 3h). Prior studies have established that Bad phosphorylation coordinates mitochondrial energy metabolism and apoptosis [22] and that nonphosphorylated Bad heterodimerizes with Bcl-xL or Bcl-2 at the mitochondrial membrane to promote cell death [23]. Figure 3h shows that phosphorylation of Bad decreased its binding with Bcl-xL; however, the binding of Bad with Bcl-xL increased dramatically with an increase in apoptosis but only in the metformin group. Taken together, these results suggest that metformin induces MSC apoptosis in an AMPK-mTOR-S6k1-Bad-dependent manner.

### Counteraction of AMPK partially restored MSC cardioprotective effects in diabetic mice with MI

**Fig. 4:**
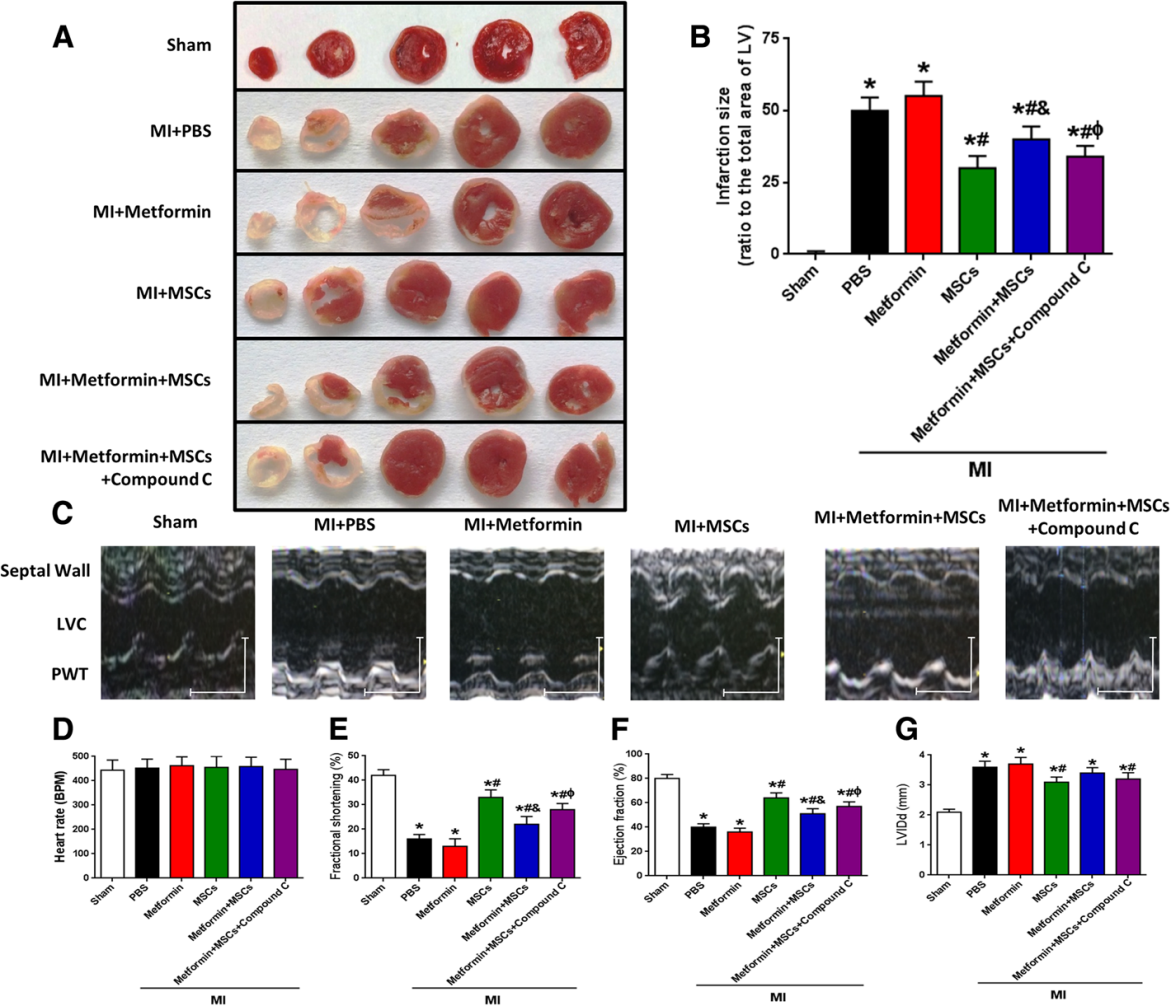
Counteraction of AMPK partially restored MSC cardioprotective effects in diabetic mice with MI. **a** Representative images of TTC-stained heart sections obtained from sham, MI + PBS, MI + metformin, MI + MSCs, MI + metformin + MSCs, and MI + metformin + MSCs + compound C groups at 4 weeks after MI. **b** LV infarct sizes expressed as the ratio of the area of the infarct band to the total area of LV (*n* = 28). **c** Representative M-mode images of hearts with sham surgery or MI at 4 weeks after PBS, metformin, MSCs, metformin + MSCs, and metformin + MSCs + compound C treatment. Scale bar: *X* axis: 0.1 s; *Y* axis: 0.2 cm. **d** Heart rates were controlled to be similar in different groups. **e**–**g** LV fraction shortening (**e**), LV ejection fraction (**f**), and diastolic left ventricle internal diameter (LVIDd, **g**) at 4 weeks after treatment (*n* = 28). *p < 0.05 vs. sham; ^#^*p* < 0.05 vs. MI + metformin; ^&^*p* < 0.05 vs. MI + MSCs; ^ɸ^*p* < 0.05 vs. MI + metformin + MSCs, by one-way ANOVA

To verify whether AMPK inhibition could protect hearts with myocardial infarction from metformin-induced MSC apoptosis in vivo, we used a myocardial infarction model in diabetic mice to evaluate the infarct size and cardiac function in post-MI hearts. Myocardial infarct size was significantly larger in diabetic mice treated with metformin + MSCs than in those treated with MSCs at 4 weeks post-MI, whereas the metformin + MSCs + compound C group showed a partially restored MSC therapeutic efficacy in MI (Fig. 4a, b). In line with the histologic change, the left ventricle EF and FS of the post-MI hearts at 4 weeks were lower in the diabetic mice treated with metformin + MSCs than in those treated with metformin + MSCs + compound C (Fig. 4e, f). In addition, MSC and metformin + compound C + MSC but not metformin + MSC transplantation significantly limited the increase in LVIDd induced by MI (Fig. 4g). These results indicate that MSC transplantation reduces the post-MI deterioration of cardiac function; this cardioprotective effect is impaired by metformin treatment in diabetic mice, and this adverse effect can be attenuated by counteraction of AMPK in diabetic mice.

### Counteraction of AMPK attenuated metformin-induced MSC apoptosis in vivo

**Fig. 5:**
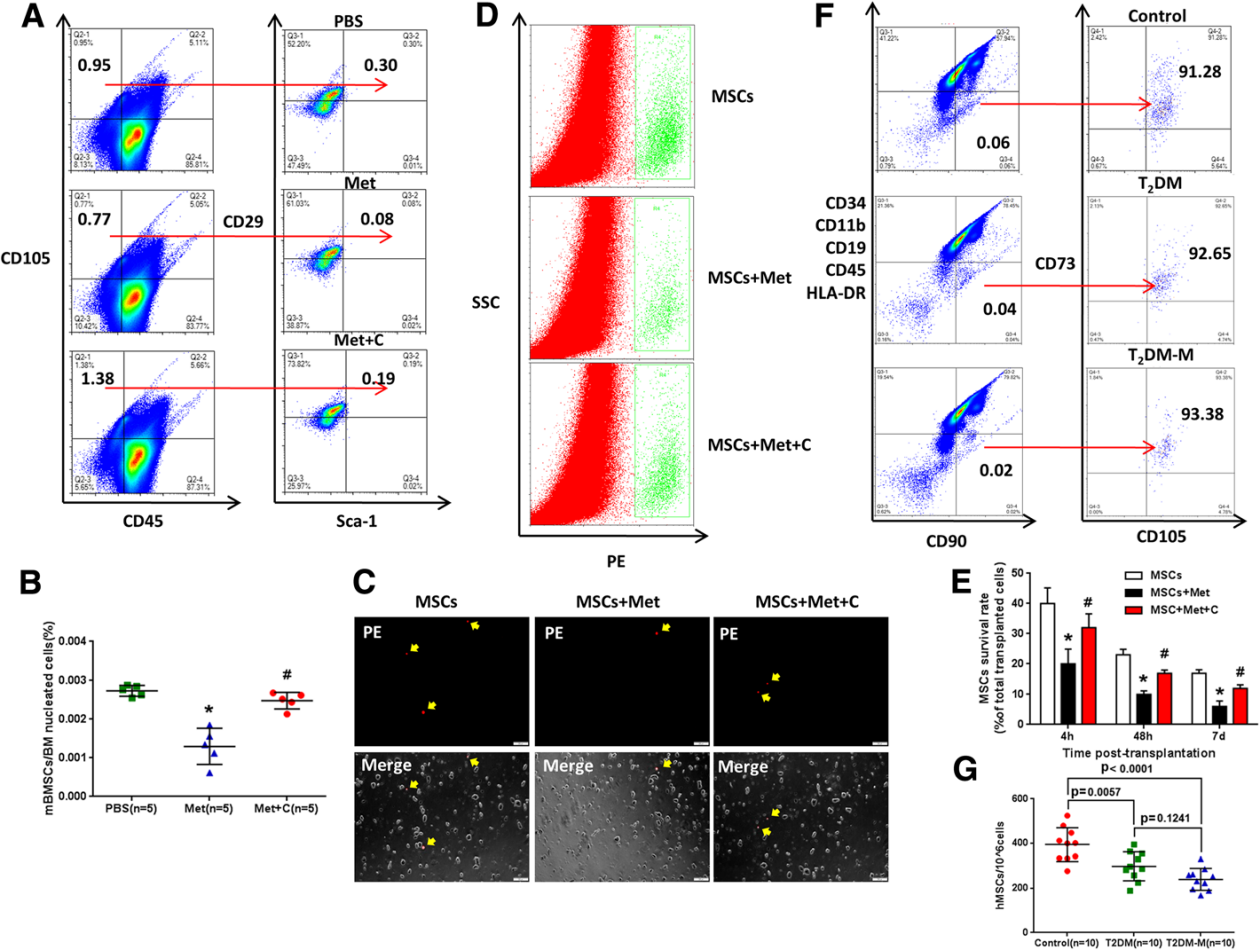
Counteraction of AMPK attenuated metformin-induced MSC apoptosis in vivo. **a** Diabetic mice were administered with PBS, metformin (250 mg/kg/day, i.g.), or metformin + compound C (0.1 mg/kg/day, i.g.) by gavage for 4 weeks, and then all mice were sacrificed to isolate mBMSCs for flow cytometry assay. **b** Metformin treatment induced a significant decrease in mBMSCs compared with PBS treatment. Compared with metformin, compound C reduced the metformin-induced mBMSC decrease (CD45−/CD105+/CD90+/Sca-1+). *p < 0.01 vs. PBS, ^#^*p* < 0.01 vs. Met, by one-way ANOVA, *n* = 5 per group. **c** Post-MI hearts with CM-DiI-labeled MSC transplantation were enzymatically digested, and small cells from the heart (< 30 μm diameter) were collected after the depletion of cardiomyocytes. As indicated with a yellow arrow, CM-DiI-labeled cells represent surviving MSCs under fluorescence microscopy. Scale bar = 100 μm. **d** Representative flow cytometric plots of surviving CM-DiI+ MSCs counted by FCM. Gate R4 indicates the CM-DiI+ cells out of all the isolated cells from the heart. **e** The percentage of surviving MSCs out of the total transplanted MSCs at different time points. *p < 0.05 vs. MSCs, ^#^*p* < 0.05 vs. MSCs + Met, by one-way ANOVA, *n* = 15 per time points. **f**, **g** Comparison among human peripheral blood MSCs (CD34−/CD11b−/CD19−/CD45−/HLA-DR−/CD90+/CD73+/CD105+) from healthy controls (control, *n* = 10), diabetic patients without metformin medication history (T_2_DM, *n* = 10), and diabetic patients with metformin medication history (T_2_DM-M, *n* = 10). Symbols represent individual subjects; horizontal lines show the mean; and data are presented as the means ± SD, statistical test applied by one-way ANOVA. Met metformin, C compound C, T_2_DM type 2 diabetes mellitus, mBMSC mouse bone marrow mesenchymal stromal cell

The in vitro data suggest that AMPK inhibition can prevent metformin-induced MSC apoptosis. Is it possible that AMPK inhibition can prevent metformin-induced MSC apoptosis in vivo? To test this hypothesis, we set up an in vivo experiment by treating diabetic mice with either metformin or metformin with compound C. After treatment with PBS, metformin (250 mg/kg/day), or metformin + compound C (0.1 mg/kg/day) decoction by gavage for 4 weeks, metformin treatment was shown to induce a significant decrease in diabetic mouse bone marrow MSCs compared with that from PBS treatment. As expected, compared with metformin alone, compound C impaired the metformin-induced mouse bone marrow MSC decrease (CD45−/CD105+/CD90+/Sca-1+) (Fig. 5a, b).

To further confirm that metformin induces MSC apoptosis in vivo, the survival of transplanted CM-DiI-labeled MSCs in MI hearts was quantified. MI hearts were digested at 4 h, 48 h, and 7 days post-transplantation. There were significantly less CM-DiI-labeled cells in the myocardium in the MSCs + metformin group than in the MSCs group at 7 days after transplantation; however, compound C reversed this effect in the MSCs + metformin + compound C group (Fig 5c, d). The better survival rate of MSCs in the MSCs and MSCs + metformin + compound C groups was confirmed with FCM analysis of isolated CM-DiI (PE+) cells at multiple time points post-transplantation (Fig. 5e).

### Metformin may display negative effects on endogenous MSCs in diabetic patients

To further characterize the effect of metformin on endogenous MSCs, we recruited 10 T_2_DM patients without metformin medication history (T_2_DM, *n* = 10), 10 T_2_DM patients with metformin medication history (T_2_DM-M), and 10 healthy volunteers (Additional file 1: Table S1) to quantify the number of peripheral blood MSCs (CD34−/CD11b−/CD19−/CD45−/HLA-DR−/CD90+/CD73+/CD105+). The mean counts of MSCs in peripheral blood of T_2_DM (297.8 ± 64.42/10^6 cells, *n* = 10) and T_2_DM-M (239.7 ± 49.08/10^6 cells, *n* = 10) patients were significantly lower than those of healthy volunteers (395.2 ± 75.61/10^6 cells, *n* = 10) (*p* = 0.0057 and *p* < 0.0001, respectively) (Fig. 5f, g). Although the number of peripheral blood MSCs in the T_2_DM-M group was lower than that in the T_2_DM group, there was no significant difference (*p* = 0.1241). This result suggests that metformin may negatively impact endogenous MSCs in diabetic patients.

## Discussion

The major cause of death and complications in T_2_DM patients is cardiovascular disease. More than 60% of all T_2_DM patients die of cardiovascular complications and an even greater percentage have serious complications. MSC-based cell therapy not only is applicable to MI but also has an antidiabetic effect in patients with diabetes [9, 24]. In addition, with the progress of aging, senescence abrogates the therapeutic potential of MSCs, so it is more necessary to use exogenous MSCs for treatment [25]. Therefore, we can foresee that in the near future, MSC-based cell therapy will be more attractive in the clinic.

MSCs are multipotent active cells with immunoregulation and tissue-repair capacities. These cells are located in virtually all postnatal organs and tissues [26]. Upon tissue damage, resident MSCs rapidly function as seed cells to help recruit a large number of MSCs from peripheral circulation to the injury site to participate in repair and regeneration [27, 28]. The efficiency of MSCs in tissue repair depends on their quality and quantity. Many studies have shown that impaired MSC quality plays a pathogenic role in diabetes [29–33], in addition to reduced numbers of MSCs [34, 35]. Therefore, we must determine not only the endogenous disease factors resulting in MSC reduction and/or dysfunction but also the impact of exogenous factors on MSCs, including various drugs involved in the treatment of diseases.

MSCs have great treatment potential for use in ischemic heart disease [9, 36]. However, a major challenge to MSC therapy is that transplanted cells undergo apoptosis. Recent studies have reported that endogenous disease factors such as myocardial infarction-related hypoxia and serum deprivation induced significant MSC apoptosis [37]. In addition, diabetes-enhanced TNF-α reduced MSC proliferation and increased MSC apoptosis [38]. Our group recently found that a decrease in circulating mesenchymal-like cells (CD34+/CD105+) is associated with the progression of chronic diabetic complications in T_2_DM [8]. Therefore, we speculate that in addition to endogenous factors including the activation of the complement system in diabetic patients recently reported by our group [8], exogenous factors, especially drugs, may have unexpected adverse effects that influence the quality and quantity of MSCs.

Many in vitro and in vivo studies have demonstrated the growth-inhibiting effects of metformin in breast, lung, endometrial, liver, medullary thyroid, and gastric cancer cell lines [39]. Several cohort studies have reported a correlation between metformin and improved cancer survival [40–43]. Moreover, metformin shows a cytotoxic effect on cancer stem cells [16]. Therefore, compared with the common cell culture additive insulin, metformin is more likely to have adverse effects on adult stem cells such as MSCs. As expected, the hypoglycemic drug used in T_2_DM, metformin, was found to dampen the MSC transplantation therapeutic effect, resulting in the impaired regeneration and repair of target tissues including the heart in an MI diabetic mouse model.

We further hypothesized that the negative effect of metformin on the therapeutic efficacy of exogenous transplanted MSCs in MI is related to MSC apoptosis. After treatment with metformin, the apoptosis of MSCs was found both in vitro and in vivo but with little effect on cardiomyocytes, endothelial cells, and fibroblasts. Specifically, metformin induces activation of AMPK-mediated apoptosis through inhibition of S6K1-Bad-Bcl-xL cell survival signaling, resulting in the upregulated expression of apoptosis-associated proteins and increased MSC apoptosis. Accordingly, the counteraction of AMPK via the AMPK inhibitor compound C or siAMPK attenuated metformin-induced apoptosis in MSCs and partially restored their cardioprotective effects in diabetic mice with MI.

Except for apoptosis, metformin may also induce senescence which has a big impact on the biology of cells. Senescence has been associated with telomere shortening and dysfunction, mesenchymal progenitor cell dysfunction, cytokine production changes, and a reduced capacity of bone marrow stromal cells to maintain functional hematopoeitic stem cells [44]. Besides, Alessio et al. reported that low-dose radiation induced senescence of human mesenchymal stromal cells and impaired the autophagy process [45]. Specifically, increased expression of pigment epithelium-derived factor in aged mesenchymal stem cells impairs their therapeutic efficacy for attenuating myocardial infarction injury [46]. Further studies should study whether metformin influences MSC’s senescence, thereby affecting its therapeutic role in the transplantation of myocardial infarction.

Although metformin caused a decrease in mouse bone marrow MSCs, metformin alone increased the infarct size of MI mouse model with no significant difference. We speculated that the possible reason is that the MSCs of the mouse itself mobilized to the site of myocardial infarction are far less than our exogenously transplanted MSCs during a short time. Furthermore, decreased peripheral blood MSCs were found in T_2_DM patients with a metformin medication history. Because of the small sample size and the small proportion of patients with T_2_DM who had no metformin medication history, there was no significant difference in the number of peripheral blood MSCs between the T_2_DM-M and T_2_DM groups.

## Conclusions

Our results highlight an unexpected adverse effect of metformin-induced MSC apoptosis through AMPK-mediated mTOR suppression that is attenuated by AMPK inhibition. Metformin was confirmed to induce apoptosis in exogenous MSCs and then affect their therapeutic efficacy. In addition, the results from animals and humans suggest that metformin has a negative effect on endogenous MSCs. Lastly, maximizing the therapeutic effects of MSC-based therapy in diabetes mellitus while minimizing the adverse effects of metformin on MSCs may be possible. Several new strategies can be considered, including taking metformin with an AMPK inhibitor, not taking metformin during MSC transplantation, or developing a modified metformin with reduced adverse effects on MSCs.

## Additional file

Additional file 1: **Table S1**. Demographic and clinical characteristics of patients. **Figure S1**. Identification of MSCs. A hUC-MSC in normal condition (40×); B osteogenic differentiation of hUC-MSC; C chondrogenic differentiation of hUC-MSC; D adipogenic differentiation of hUC-MSC; E detection of surface markers of hUC-MSC by flow cytometry. **Figure S2**. Metformin has no effect on mouse cardiomyocytes, human endothelial cells, and human fibroblast survival. A and B, the ratio of mouse cardiomyocyte apoptosis (annexin V+) induced by 2 mM metformin at 24 h, 48 h, and 72 h; C and D, the ratio of human endothelial cells apoptosis (annexin V+) induced by 2 mM metformin at 24 h, 48 h, and 72 h; E and F, the ratio of human fibroblast apoptosis (annexin V+) induced by 2 mM metformin at 24 h, 48 h, and 72 h. Bars in B, D, and F represent the mean ± SEM (*n* = 3 per group). Statistical test applied by one-way ANOVA. (DOCX 11562 kb)

## Abbreviations

AMPK: AMP-activated protein kinase
hUC-MSCs: Human umbilical cord-derived mesenchymal stromal cells
LAD: Left anterior descending
MIs: Myocardial infarctions
MSCs: Mesenchymal stromal cells
T_2_DM: Type 2 diabetes mellitus
