# Supplementary material for: Metformin induces apoptosis in mesenchymal stromal cells and dampens their therapeutic efficacy in infarcted myocardium
Source: Stem Cell Res Ther. 2018 Nov 8;9:306. doi: 10.1186/s13287-018-1057-0 (PMC6225675; doi:10.1186/s13287-018-1057-0)
Supplement: Supplementary file 1 — Table S1. Demographic and clinical characteristics of patients. Figure S1. Identification of MSCs. A hUC-MSC in normal condition (40×); B osteogenic differentiation of hUC-MSC; C chondrogenic differentiation of hUC-MSC; D adipogenic differentiation of hUC-MSC; E detection of surface markers of hUC-MSC by flow cytometry. Figure S2. Metformin has no effect on mouse cardiomyocytes, human endothelial cells, and human fibroblast survival. A and B, the ratio of mouse cardiomyocyte apoptosis (annexin V+) induced by 2 mM metformin at 24 h, 48 h, and 72 h; C and D, the ratio of human endothelial cells apoptosis (annexin V+) induced by 2 mM metformin at 24 h, 48 h, and 72 h; E and F, the ratio of human fibroblast apoptosis (annexin V+) induced by 2 mM metformin at 24 h, 48 h, and 72 h. Bars in B, D, and F represent the mean ± SEM (n = 3 per group). Statistical test applied by one-way ANOVA. (DOCX 11562 kb) [file 13287_2018_1057_MOESM1_ESM.docx]

**Supplementary Data**

Table S1. Demographic and Clinical Characteristics of Patients.

|  | T_2_DM | T_2_DM-M | Healthy Volunteers |
| --- | --- | --- | --- |
| Patients no. | 10 | 10 | 10 |
| Male no. (%) | 8 (80) | 6(60) | 8 (80) |
| Mean age (year) | 64 | 62.32 | 61.11 |
| BMI (kg/m^2)^ | 25.6±1.3* | 26.2±1.2* | 21.3±1.6 |
| Mean duration of disease (year) | 9.27±3.18 | 12.15±2.35 |  |
| Mean medication(Metformin) (year) |  | 8.69±1.77 |  |
| FPG (mmol/L) | 9.6±1.9** | 9.4±1.7** | 4.8±0.7 |
| HbA1c (%) | 8.2±1.2** | 8.1±1.4** | 4.5±0.6 |
| Diabetes-related complications no. (%) | |  |  |
| Diabetic Foot | 1 (10) | 2 (20) |  |
| Diabetic nephropathy | 3 (30) | 3 (30) |  |
| Diabetic peripheral neuropathy | 7 (70) | 6 (60) |  |
| Diabetic retinopathy | 3 (30) | 4 (40) |  |
| Diabetic ketoacidosis | 1 (10) | 1 (10) |  |

T_2_DM: type 2 diabetes mellitus. BMI: body mass index. FPG: fasting plasma glucose. Data are presented as mean±SD. P-values were obtained by ANOVA. *=P<0.05. **=P<0.01. All comparisons were against Healthy Volunteers.


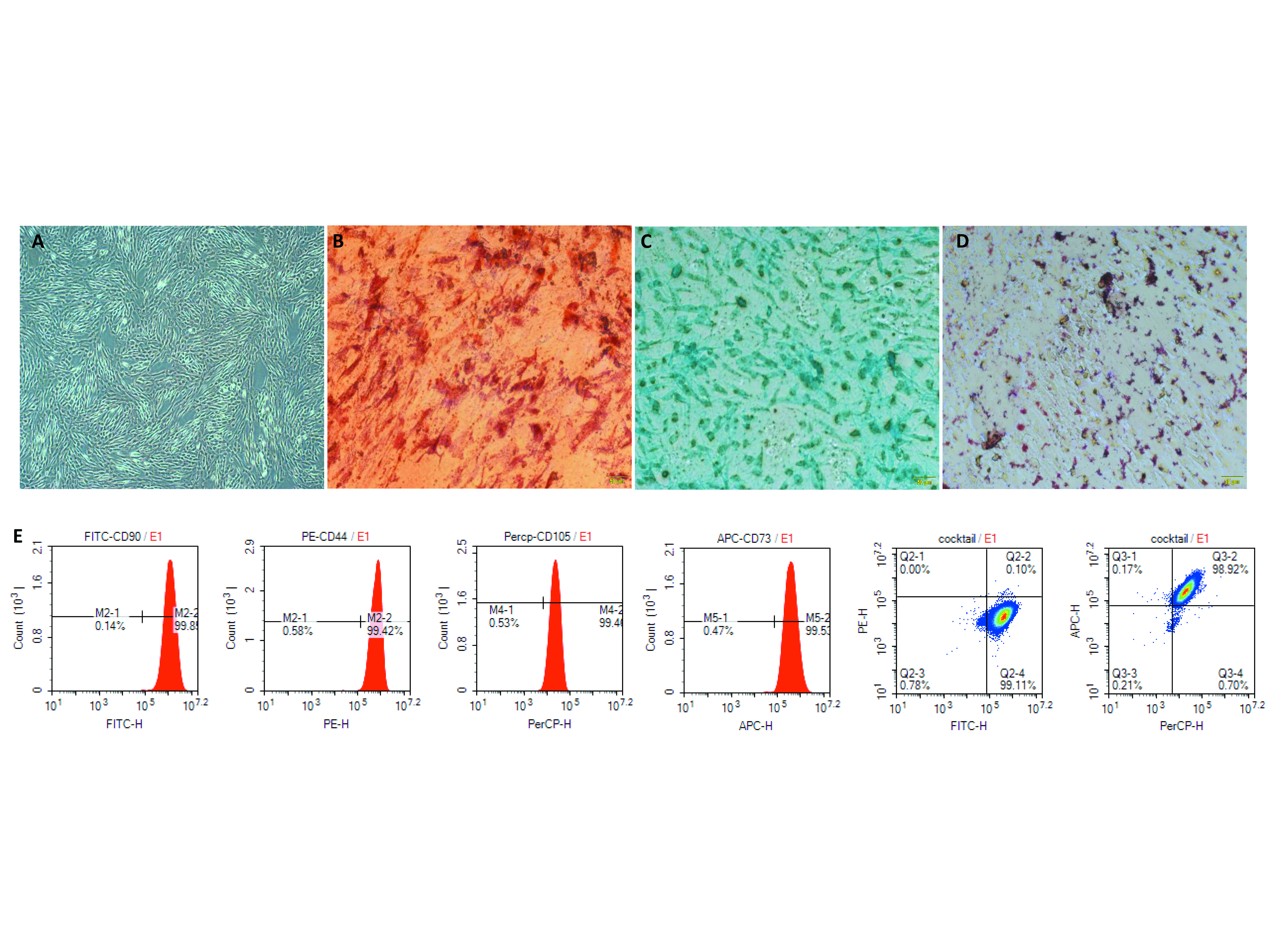


**Figure S1**. Identification of MSCs. **A** hUC-MSC in normal condition （40×）； **B** Osteogenic differentiation of hUC-MSC；**C** Chondrognic differentiation of hUC-MSC；D. Adipogenic differentiation of hUC-MSC；E. Detection of surface markers of hUC-MSC by flow cytometry.


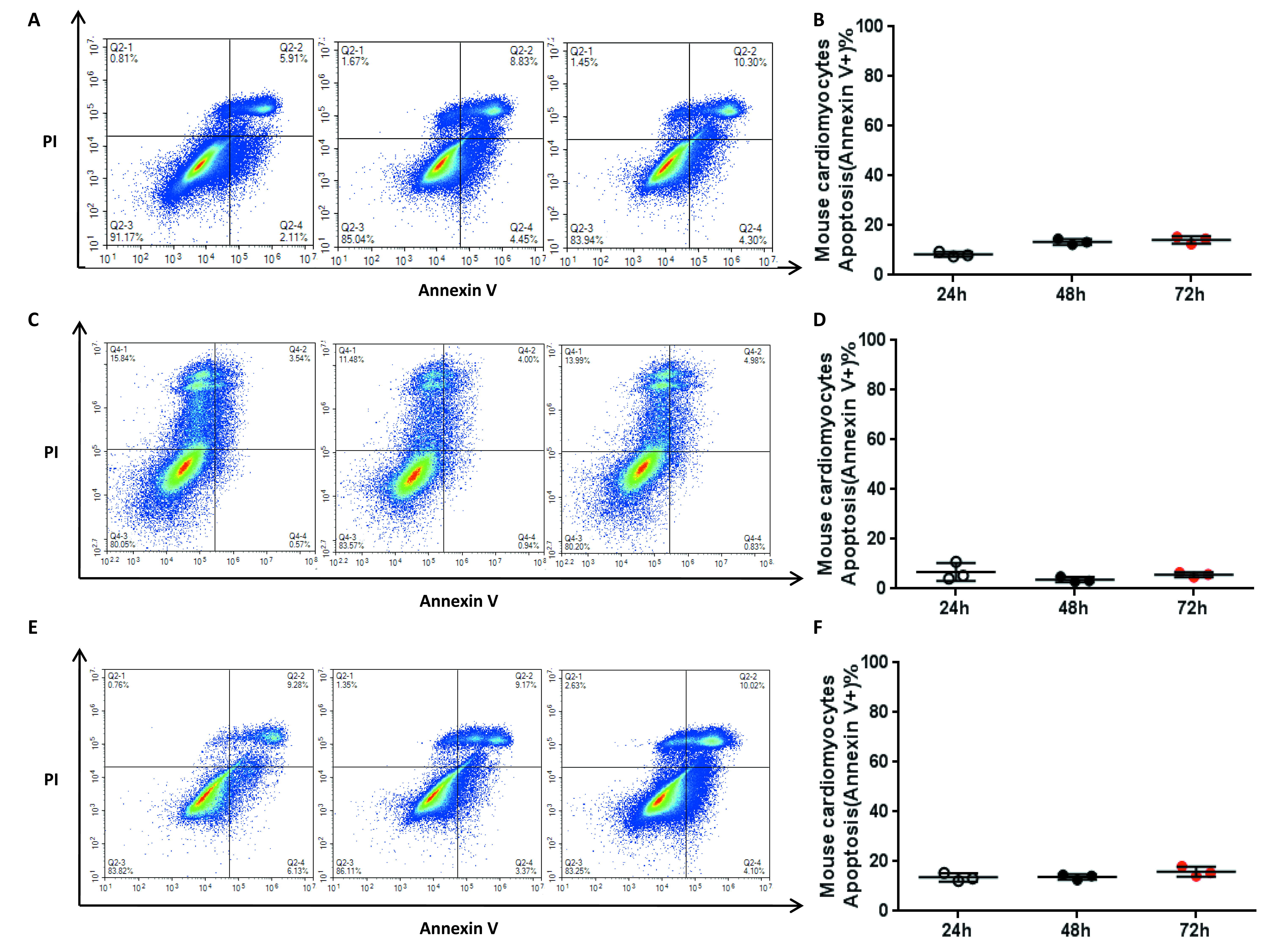


**Figure S2**. Metformin has no effect on mouse cardiomyocytes, human endothelial cells and human fibroblasts survival. **A** and **B**, The ratio of mouse cardiomyocytes apoptosis (Annexin V+) induced by 2 mM metformin at 24h, 48h, 72h; **C** and **D**, The ratio of human endothelial cells apoptosis (Annexin V+) induced by 2 mM metformin at 24h, 48h, 72h; **E** and **F**, The ratio of human fibroblasts apoptosis (Annexin V+) induced by 2 mM metformin at 24h, 48h, 72h. Bars in **B** , **D** and **F** represent the mean±SEM (n=3 per group). statistical test applied by one-way ANOVA.
